# Do ENSO and Coastal Development Enhance Coastal Burial of Terrestrial Carbon?

**DOI:** 10.1371/journal.pone.0145136

**Published:** 2015-12-21

**Authors:** Peter I. Macreadie, Timothy C. Rolph, Ron Boyd, Claudia J. Schröder-Adams, Charles G. Skilbeck

**Affiliations:** 1 Plant Functional Biology and Climate Change Cluster, University of Technology Sydney, Broadway, New South Wales 2007, Australia; 2 Centre for Integrative Ecology, School of Life and Environmental Sciences, Deakin University, Burwood, Victoria 3125, Australia; 3 School of Geosciences, University of Newcastle, Callaghan 2308, Australia; 4 Department of Earth Sciences, Carleton University, Ottawa, Ontario K1S 5B6, Canada; 5 School of the Environment, University of Technology Sydney, Broadway, New South Wales 2007, Australia; Griffith University, AUSTRALIA

## Abstract

Carbon cycling on the east coast of Australia has the potential to be strongly affected by El Niño-Southern Oscillation (ENSO) intensification and coastal development (industrialization and urbanization). We performed paleoreconstructions of estuarine sediments from a seagrass-dominated estuary on the east coast of Australia (Tuggerah Lake, New South Wales) to test the hypothesis that millennial-scale ENSO intensification and European settlement in Australia have increased the transfer of organic carbon from land into coastal waters. Our data show that carbon accumulation rates within coastal sediments increased significantly during periods of maximum millennial-scale ENSO intensity (“super-ENSO”) and coastal development. We suggest that ENSO and coastal development destabilize and liberate terrestrial soil carbon, which, during rainfall events (e.g., La Niña), washes into estuaries and becomes trapped and buried by coastal vegetation (seagrass in this case). Indeed, periods of high carbon burial were generally characterized as having rapid sedimentation rates, higher content of fine-grained sediments, and increased content of wood and charcoal fragments. These results, though preliminary, suggest that coastal development and ENSO intensification—both of which are predicted to increase over the coming century—can enhance capture and burial of terrestrial carbon by coastal ecosystems. These findings have important relevance for current efforts to build an understanding of terrestrial-marine carbon connectivity into global carbon budgets.

## Introduction

Coastal areas dominated by seagrasses, salt marshes, and mangroves—referred to as ‘blue carbon’ habitats—are among the most powerful carbon (C) sinks on the earth. They occupy only a fraction (0.8–2.6%) of the total global area of terrestrial forests (temperate, tropical, boreal), yet because of their efficient C sequestration rate (42-times higher than terrestrial forests) and long-term burial, they are likely to exceed global C capture and storage by terrestrial forests [1]. However, much of the C that is buried by coastal areas is not produced by blue C habitats themselves, but instead originates from terrestrial ecosystems [2, 3]. Because blue C habitats are found at the interface between land and sea, they are in prime position for capturing terrestrial C that runs off the land. Therefore, environmental processes that facilitate terrestrial C production and runoff—such as the El Niño-Southern Oscillation (ENSO)–can be expected to increase C burial within nearby coastal ecosystems.

ENSO directly affects the climate of the Pacific, manifesting as swings between La Niña and El Niño conditions. During El Niño events, the Pacific trade winds that normally flow in a westerly direction slacken, cause warming of the central and eastern tropical Pacific. On the east coast of Australia, El Niño is associated with low rainfall, drought, and fire, whereas during La Niña the pattern is reversed, and eastern Australia experiences high rainfall, flooding, and high terrestrial primary productivity. ENSO-driven drought-flood cycles therefore has the overall effect of destabilising and mobilising terrestrial C [4], and could have disproportionately large impacts on C budgets [5]–i.e. El Niño conditions destabilize and liberate terrestrial soil C which accumulates on the land; La Niña rainfalls then wash this terrestrial C into estuaries where it becomes trapped and buried by coastal vegetation. For example, in the western Pacific region its been shown that major, episodic hydrologic events can account for 77–92% of land C transfer into coastal waters over decadal time scales [6]. Such events increase C supply and also cause C to bypass ‘normal’ C remineralisation processes by reducing C transport times [7], and should therefore increase total C burial within coastal waters that receive terrestrial C runoff. Empirical evidence of such terrestrial-coastal C feedbacks should be evident within paleo-ENSO records, but remain to be demonstrated.

We investigated the effects of past millennial-scale ENSO activity on coastal C burial using paleoreconstructions of sediments extracted from blue C habitats in eastern Australia. We examined multiple proxies (carbon stable isotopes, benthic foraminifera, sedimentation rate, and macro charcoal deposits) within sediments covering the past 9000 years. Importantly, this duration included a period (~1000–3000 cal. yrs BP) when ENSO intensity increased dramatically within the western Pacific [8], causing millennial-scale ENSO intensification known as ‘super-ENSO’. Paleoreconstructions are necessary to develop a long-term perspective because instrumental records of ENSO behaviour only cover the past 150 years and, because C stocks change slowly, they require a long time (decades-millennia) perspective. The study region (eastern Australia), much like the rest of the western Pacific, has a drought, fire, and flood regime that is influenced by long-term ENSO cycles [9, 10].

## Materials and Methods

**Fig 1:**
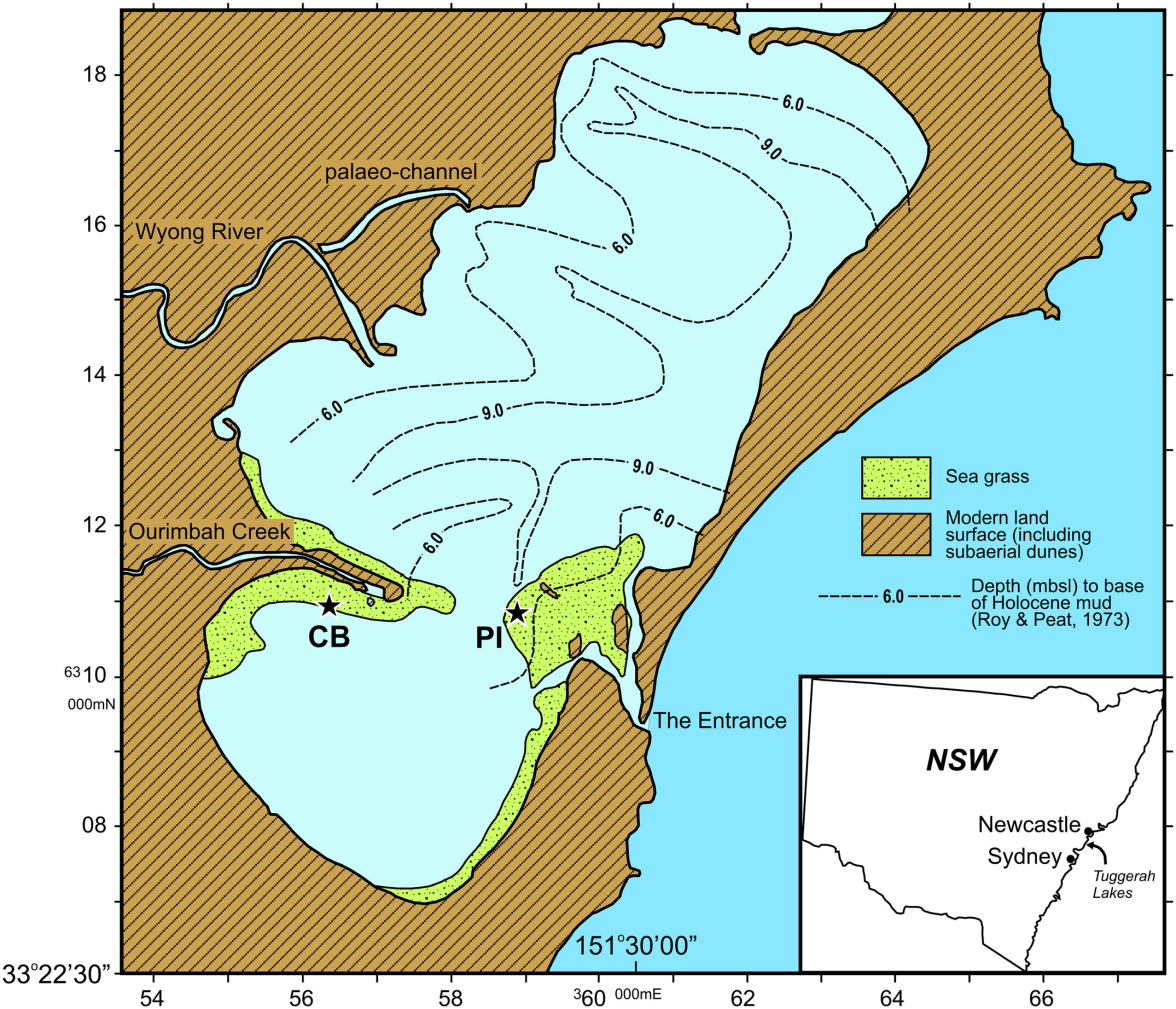
Map of Tuggerah Lake (NSW, Australia) showing the two study site locations (starred) where sediment cores were taken; Chittaway Bay (CB) and Pelican Island (PI).

Sediment cores (n = 1 per site) were recovered from two sites—Pelican Island (PI) and Chittaway Bay (CB)–within Tuggerah Lake on the Australian east-coast estuary (Fig 1). Maritime services were notified of the core collection; however, as the cores were extracted from a public area of the waterway where there were no endangered or protected species, no specific permission or permitting was required. The Lake is a shallow (max depth <4 m) barrier estuary, which lies behind a Holocene and Pleistocene coastal sand barrier complex [11]. Freshwater enters the lake mainly from Ourimbah Creek and the Wyong River. Seagrass beds, mangroves, and saltmarsh are distributed around the margins of the lake.

Cores were collected in 90 mm diameter, 6 m long plastic tubes, pushed into the sediment using a hammer system. At site PI the core barrel penetrated to 434 cm (534 cm below sea level) before meeting excessive resistance associated with penetration of Pleistocene stiff clay. At site CB, only 251 cm of sediment penetration was achieved (331 cm below sea level), reflecting the shallower location of the Holocene-Pleistocene boundary at this site. Cores were returned to the laboratory (University of Technology Sydney) where they were split vertically for sub-sampling and lithological description. Subsequently, cores were stored frozen to minimise sediment alteration.

Total organic C (TOC) was measured via loss on ignition (LOI). Samples were heated in two stages (105°C and 550°C) and weighed following each heating. TOC content was obtained by dividing weight lost between 105°C and 550°C by 2.5; the proportion of water:C in organic matter (CH_2_O). Organic matter source variability was investigated by measuring δ_13_C (via isotope-ratio mass spectrometry) from the lower two units of the PI core.

Grain size analysis was performed using a Malvern Mastersizer/E set on the 1.2–600 μm range. Samples were washed with a hydrogen peroxide solution to disperse faecal pellets. Larger shell and organic fragments were removed. Samples were washed through a 500 μm sieve into the Mastersizer dispersion tank. Coarser sediment was dried and further characterised using 707 μm and 1000 μm sieves, with data then combined with the Mastersizer results.

Samples for foraminiferal analysis were taken every 20 cm in both cores and were washed through a 63 μm sieve; the CB core, an additional sample was collected at 190 cm, while the 220 cm sample was not collected because it coincided with a large shell layer. Foraminiferal specimens were then picked from dried residues and identified.

Conventional and AMS radiocarbon dating was carried out at the Waikato Carbon Dating Laboratory and at the ANSTO AMS facility at Lucas Heights, Sydney. Conventional dates from bulk mud samples collected at lithological and magnetic boundaries were supplemented by twelve AMS^14^C dates on whole shells and wood/charcoal fragments. Calibration of the radiocarbon dates was performed with Oxcal3.10 using the calibration curves shCal04.14c for the wood and charcoal samples and Marine04.14c for the shell samples; in the latter case, a local correction of 3 ± 70 was applied.

Raw data can be found at http://dx.doi.org/10.6084/m9.figshare.1609800

## Results and Discussion

**Fig 2:**
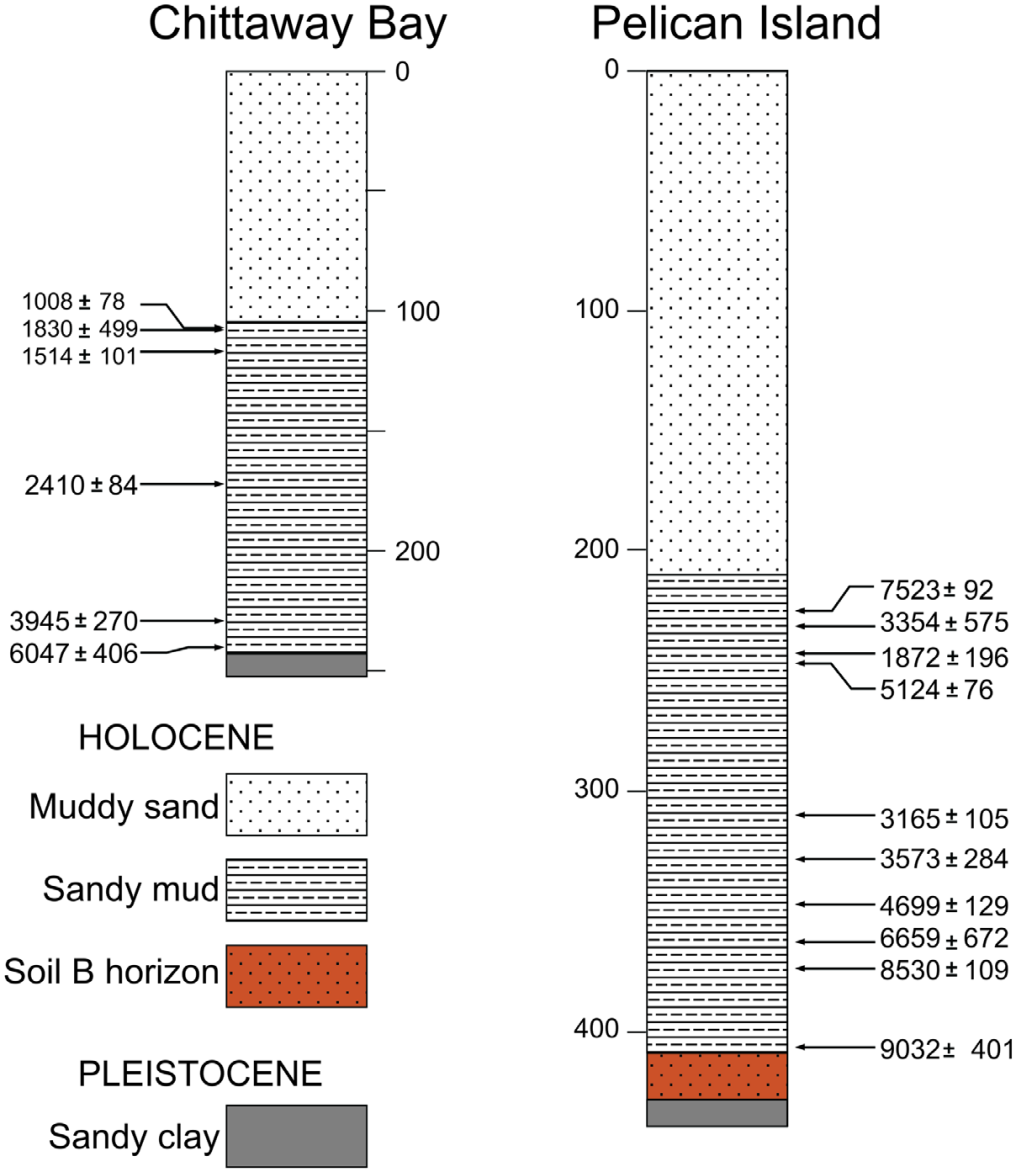
Sediment cores taken from Chittaway Bay and Pelican Island showing how sediment type changes with depth (cm) down core and age (^14^C data, mean ± SE).

Cores used for the study have a 3-unit Holocene lithostratigraphy (Fig 2), consisting of a basal clay unit (unit 3), with an erosional upper surface, overlain by a sandy mud unit (unit 2) that grade into an upper muddy sand unit (unit 1). The Pelican Island core has a partial soil B horizon, with organic fragments that may be root material. Small, sand-sized fragments of charcoal are dispersed throughout, with occasional larger fragments. The top of the core contains sea-grass fragments, roots and increased mud. In both cores, initial sedimentation following the unconformity is central basin mud facies, suggesting formation of a seaward barrier immediately following stabilization of the high-stand sea level, or transgression of relict Pleistocene barriers [12].

**Fig 3:**
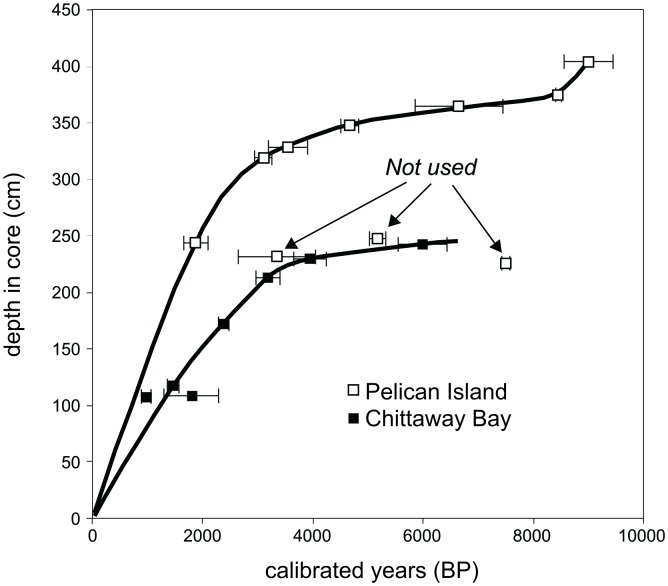
Age-models of sediment accumulation rates.

Sediment accumulation rates ranged from 0.1–0.5 mm yr^-1^ for sandy and 1.2–1.9 mm yr^-1^ for mud units (Figs 2 and 3). For the Pelican Island site, three samples from the interval 230–250 cm gave inconsistent results; a mud-derived date and two charcoal-derived dates are older than dates that were obtained from material located significantly deeper (90 cm or more) in the same core. Conversely, the age obtained from a small disarticulated *Notospisula* shell collected at 243 cm is stratigraphically consistent with the dates obtained from below 300 cm. The shell was not associated with visible bioturbation features but may be younger than the enclosing sediment. Grain-size and geochemical data indicate the growing influence of marine sand in the upper part of unit 2, reflecting the arrival of flood-tide delta sand at the site, so it is possible that reworking of flood-tidal-delta sand could have delivered old charcoal to the Pelican Island site, affecting dates obtained from charcoal fragments or from mud samples.

**Fig 4:**
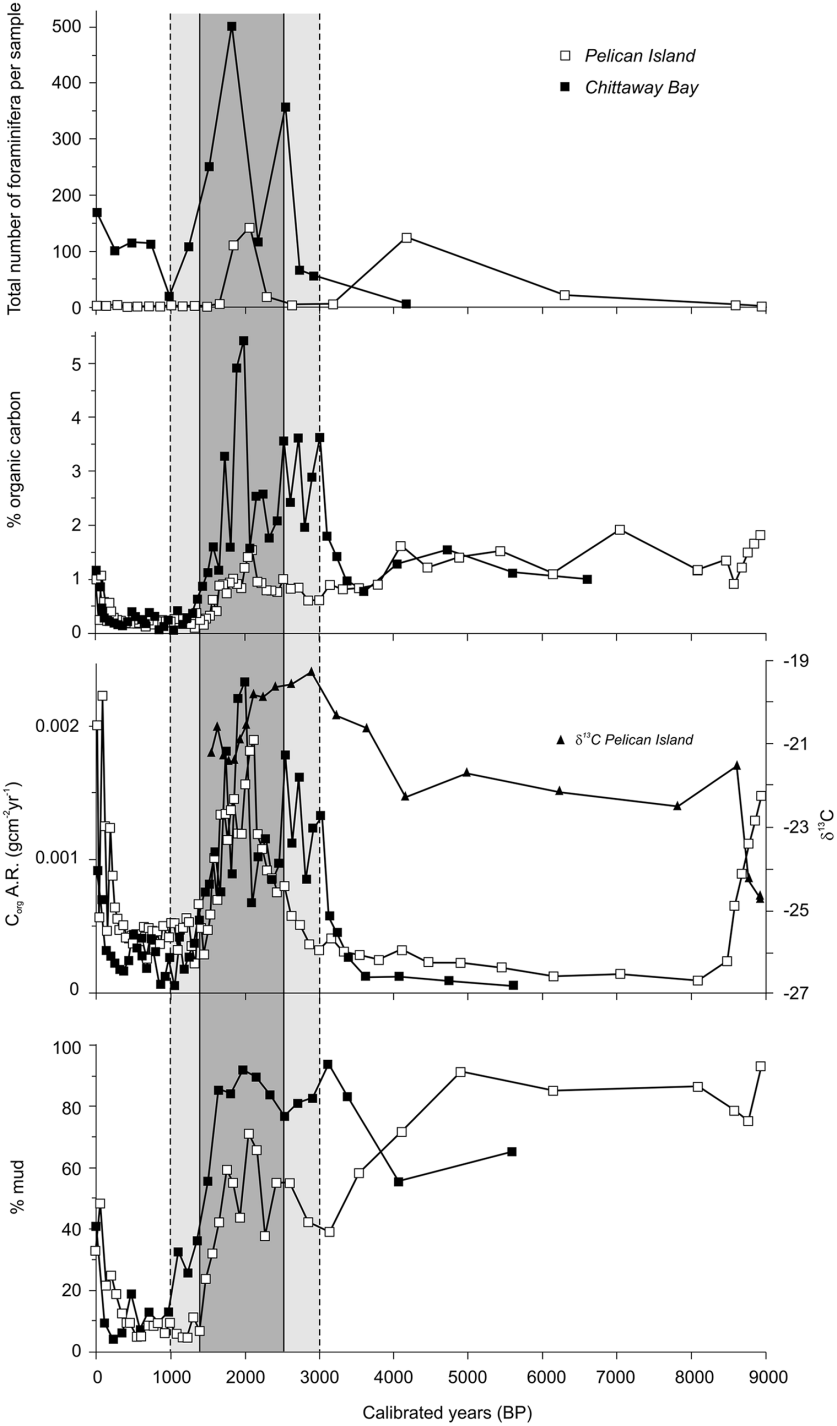
Sediment archives showing geochemical changes during the past 9000 years. Increased foraminifera abundance, % organic carbon (C), organic C accumulation rate (C_org_ A.R.), and % mud are observed during ENSO intensification (grey-shaded area) and since European settlement (~200 years ago). Darker shading indicates the peak period following re-establishment of ENSO after the mid-Holocene hiatus (Gagan et al. 2004). Sediment cores taken from Tuggerah Lake, NSW, Australia (Sites: PI—Pelican Island, CB—Chittaway Bay).

**Fig 5:**
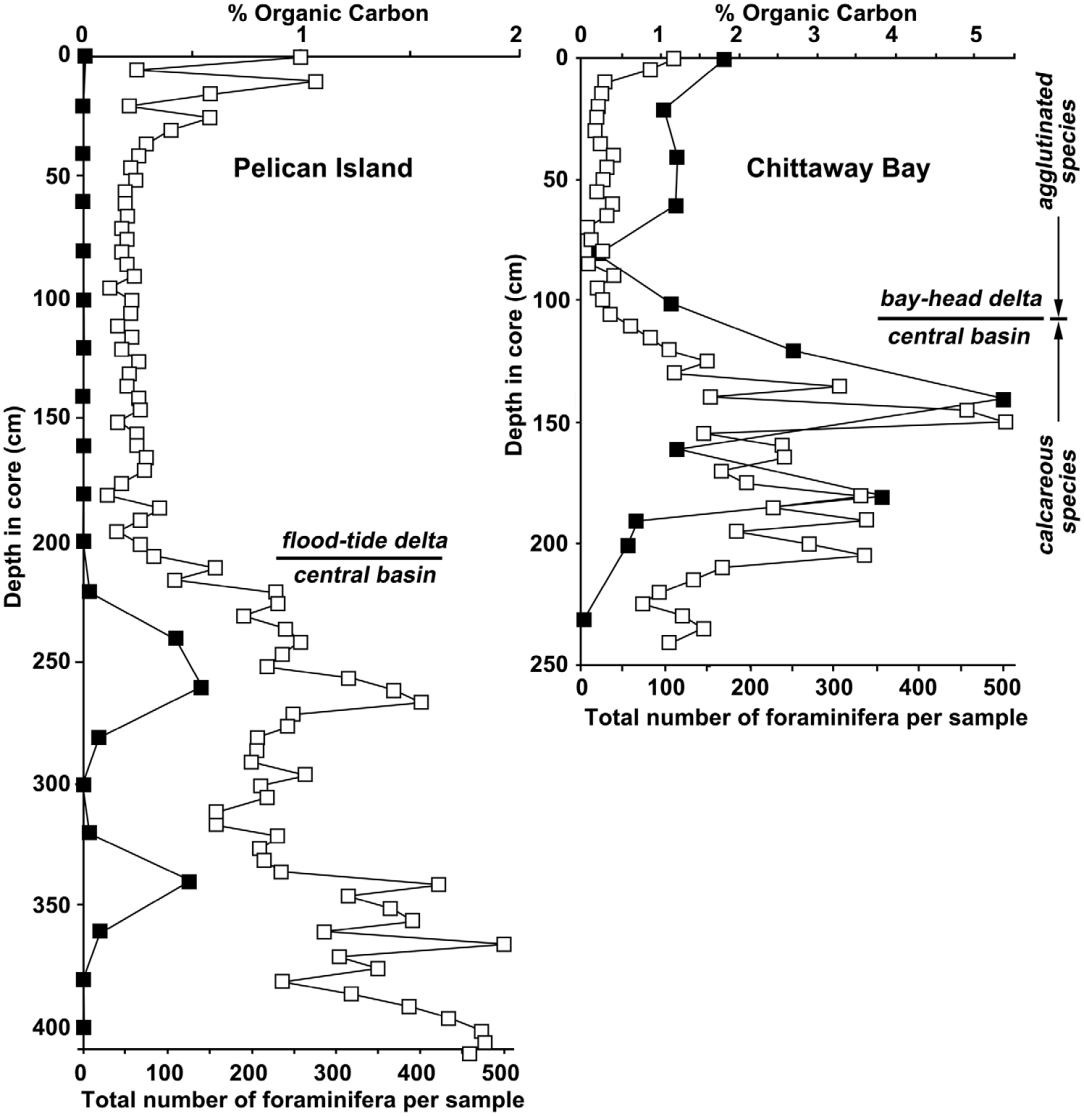
Downcore variation in foraminiferal abundance together with % organic carbon.

Surface sediments show an increase in muddy sand content, along with apparent increases in benthic foraminifera abundances/preservation and C content (Figs 4 and 5). Though recent (surface) sediments have not been aged, the timing of these changes are roughly consistent with the timing of European settlement (1788) and industrial development (i.e. the past ~200 years); both of which have resulted in increased nutrient loadings that might have stimulated foraminifera populations, and increased sediment runoff due to forest clearing that would have increased both the % mud and potentially C input [13, 14].

Foraminiferal faunas consisted of benthic species and show low species richness. The estuarine sub-environments are inferred from the lithostratigraphy and the grain size data. The assemblage of the Pelican Island core is dominated by calcareous species. The sands of the floodtide delta in the Pelican Island core are nearly barren of foraminifera, while the bay-head delta assemblage of Chittaway Bay is dominated by agglutinated taxa, of which *Tritaxia conica* is most common. In both cores the highest abundances occur within the finer sediments of unit 2; finer grain-size leads to a greater retention of organic matter, a significant factor for these detritus feeders. The unit 2 sediments contain mainly calcareous species, dominated by *Ammonia*, a typical lagoonal and estuarine indicator taxon that tolerates lowered salinities.

Regional climate indicators are consistent with evidence for a major change in the ENSO climate system; the onset of modern ENSO periodicities occurs at ~5000 years BP [8, 15], and subsequently, ENSO amplitude increased abruptly at ~3000 cal. years BP, reaching a maximum at ~2000 cal. years BP [8]. During periods of maximum ENSO intensity (‘super-ENSO’; ~1000–3000 cal. yrs BP) [8], several major changes occurred (Fig 4): (1) the abundance of foraminifera increased; (2) % C content and organic C accumulation rate (C_org_ A.R.) rose; and (3) % mud content increased. Furthermore, sediment accumulation increased after ~3000 cal. years BP (Fig 3), which would likely have enhanced preservation of C due to rapid burial and removal from aerobic degradation. Signs of dissolution in calcareous foraminiferal tests were observed, a factor also present in central muddy basin sediments of Port Stephens, NSW [16]. Increased amounts of organic matter in an oxygenated environment ultimately results in greater production of carbonic acid dissolving calcareous tests [17]. Since the accumulation rate of the fine-grained clastic component will influence the C content, and since the latter is related to factors such as primary productivity and redox processes, we have used the sediment accumulation rate, water content, and total organic C (%TOC) data to estimate the organic C accumulation rate (C_org_ A.R.; Fig 4). C_org_ A.R. data show that recent sediments (corresponding with the approximate timing of European settlement in Australia—but note that recent sediments have not been aged) there has been a substantial increase in coastal C burial, supporting recent suggestions by Regnier et al. [18] that industrialisation has increased the amount of C transfer from terrestrial to coastal ecosystems.

**Fig 6:**
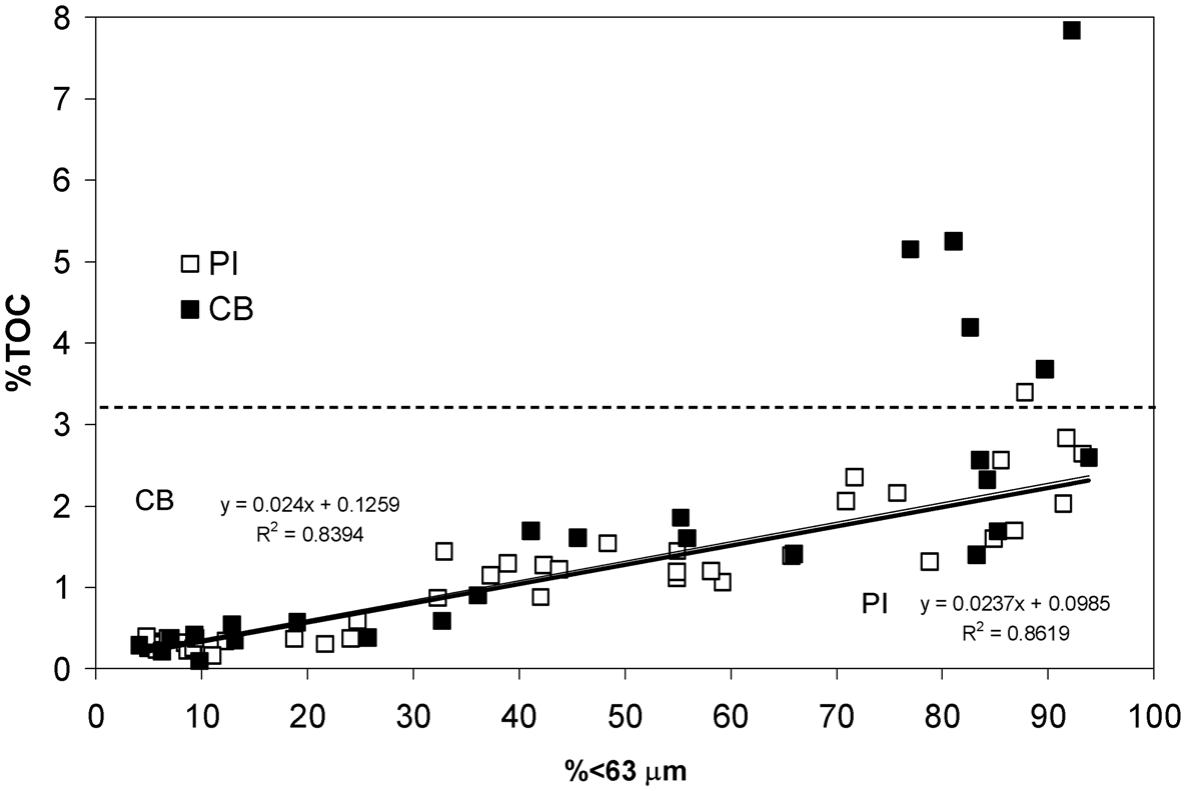
Relationship between sediment % total organic carbon (%TOC) and mud content. Data taken from the <63 μm fraction from unit 1 and 2 sediments (Fig 2).

The % C data show a strong correlation with mud content, with both cores having near identical linear regression coefficients for samples with C values <2% (Fig 6). This result is consistent with findings that C content is strongly linked to sorption-preservation of labile C on mineral surfaces [19]. We suggest that deviations of data points from the linear trend (i.e. samples with >2% C) are the result of changes in the efficiency of degradation processes and fluctuations in the input of refractory terrestrial C; the latter is more resistant to microbial degradation [20]. Indeed, the data with C >2% are all from within a distinct interval of enhanced C content at 150–200 cm (Fig 4); this corresponds with the interval within which we observed an increased content of wood and charcoal fragments, and is interpreted as the period of ENSO intensification (although there is no way to differentiate this from aboriginal burning practices during the period of cultural intensification during the late Holocene). Furthermore, stable isotope analyses (δ^13^C) of selected samples suggest that C sources are changing during this period [2, 21] (Fig 4).

The lack of any correspondence in mud percent, foraminiferal abundance, and organic C content for the early Holocene (<3000 cal yr. BP; Figs 4 and 5) is likely due to the differing local depositional environmental conditions at the time—i.e. during the early Holocene mud deposition cannot be directly tied to a local fluvial source (Macreadie et al. 2015).

There is evidence that the patterns of ENSO-induced transfer of terrestrial C into coastal zones observed here also apply to other ENSO-affected areas, including the eastern-Pacific. Enhanced coastal C burial has been recorded in coastal sediments of Puerto Rico due to enhanced runoff around 1300–1500 cal. years BP [22]. Similarly, La Niña flood events were the cause of thick episodic layering of C within Andean-Amazonian foreland sediments, attributed to land runoff and rapid sedimentation rates [23]. We argue that transfer of C from terrestrial ecosystems and subsequent burial within the coastal zone will occur anywhere that ENSO causes drought-flood events, so long as there are blue C habitats to capture C runoff before it can be advected to the open ocean. In a separate study from the same site [11], we found little evidence of changes in relative sea level that could account for any major changes in the storage of C.

An advantage of transferring terrestrial C into coastal waters is that the C is likely to be bound over longer time-scales. Blue C habitats, which occur on every continent except Antarctica, can bury C over millennia (as demonstrated in Fig 4), whereas most terrestrial systems sequester C over considerably shorter time-scales [1, 24]. In addition, blue C habitats have much higher C burial efficiencies than most terrestrial habitats [1]. There are two caveats to this suggestion. The first is that it is contingent upon the maintenance of blue C habitats, which are currently facing significant global decline (~20–50% loss) [1], and could shift from C sinks to C sources, thereby accelerating climate change. Annual emissions from loss of estuarine blue C habitats are estimated at 0.15–1.02 Pg (billion tons), which is equivalent to 3–19% of those from deforestation [25]. Second, it assumes that terrestrial C transfer occurs primarily in the form of sequesterable particulate C, rather than being released directly into the atmosphere as CO_2_ via burning or remineralisation [26].

Of the 9.1 Pg C yr^-1^ (1 Pg C = 1 petagram = 10^9^ metric tons of C) that was emitted anthropogenically during 2000–06, it estimated that 45% accumulated in the atmosphere, 31% was sequestered within terrestrial ecosystems, and the remaining 24% was bound within marine habitats [27]. The marine burial component is the most poorly constrained estimate, and it does not take into consideration that a substantial proportion of marine-sequestered C may be of terrestrial origin, which increases the risk of ‘double-counting’ during C budgeting. Translocation of C from one system to another does not result in an increase in net sequestration, just a redistribution of previously-sequestered C. Understanding the dynamics of this C redistribution process is critical for managing C sequestration to help mitigate climate change, especially as climate change itself causes an increase in the frequency and intensity of extreme weather events [28].

Future consolidation of our findings should include: (1) age dating for recent sediments (Pb210) to confirm the timing of European settlement; (2) additional radiocarbon dates around the time of super-ENSO to refine the age model; (3) analysis of pollen and ostracods to support the interpretation of the foraminiferal data; and (4) more cores from the center of the lake, as well as from other ENSO-affected estuaries in the region. Furthermore, another constraint in the interpretation of our results (one that affects all studies on C stocks) is that the apparent increase in surface C stocks due to anthropogenic activities may not necessarily lead to more C being sequestered long-term. This is because surface sediments are more vulnerable to remineralisation, meaning that only a fraction will become preserved and enter the long-term sedimentary C pool.

In summary, this study suggests that C burial within the coastal zone is linked with terrestrial responses, and that ENSO intensification increases coastal C burial at the expense of terrestrial C losses. The high burial efficiency and long-term burial capacity of coastal habitats leads us to conclude that ENSO-driven transfer of terrestrial C into coastal zones may increase overall C storage in ENSO-affected areas. Furthermore, our results show that anthropogenic activities in the catchment are triggering C transfer events equivalent to ‘Super-ENSO’. These results have important implications for new models that predict increases in ENSO intensification with future climate change over the coming century [29]. We conclude that ENSO intensification and coastal development—which are predicted to increase over the coming century—may enhance coastal burial of terrestrial C.
